# A single epigenetic repressor controls opposing developmental tempos in human neural and cranial neural crest lineages

**DOI:** 10.64898/2026.10.09.758088

**Published:** 2026-10-13

**Authors:** Zeynep Eda Atmaca Duran, Marcella Birtele, Shweta Mendiratta, Ramesh Yelgandula, Claire Stevens, Suhn Rhie, Giorgia Quadrato, Oliver Bell

## Abstract

The precise timing of lineage specification is essential for embryonic development, yet the epigenetic mechanisms enforcing human developmental tempo remain poorly understood ^1,2^ . Heterozygous loss-of-function variants in *ZNF462* cause Weiss-Kruszka syndrome (WKS), a pleiotropic disorder characterized by neurodevelopmental delays and craniofacial dysmorphisms ^3–5^ . Here, we show that ZNF462 acts as an epigenetic gatekeeper that coordinates human developmental tempo across divergent lineages by repressing cell lineage-specific gene enhancers. In human embryonic stem cells (hESCs), ZNF462 maintains self-renewal by recruiting the G9A/GLP histone methyltransferase complex to silence primate-specific retrotransposon-derived enhancers. Upon neural lineage commitment, ZNF462 redirects to hominoid-specific LTR5_Hs elements and divergent lineage-specific enhancer programs, where its loss produces opposing kinetics. In neural progenitor cells (NPCs), ZNF462 enforces G9A/GLP-dependent repression of ZIC-driven neural enhancers, and loss of this repression traps NPCs in a progenitor state, slowing cortical neurogenesis without impairing terminal neurogenic capacity. In neural crest cells (NCCs), ZNF462 targets repressive chromatin to silence SOX9 family-driven enhancers. Here, ZNF462 haploinsufficiency triggers premature activation of definitive NCC specifiers, accelerating differentiation. These findings establish that a single repressor can simultaneously decelerate and accelerate developmental tempo by gating lineage-specific enhancers, placing Weiss-Kruszka syndrome into the emerging class of human developmental timing disorders.

